# Values and their relationship with emotion processing and physical and psychological symptoms among Jewish and Arab breast cancer survivors

**DOI:** 10.3389/fpsyg.2023.1297377

**Published:** 2024-01-04

**Authors:** Maimounah Hebi, Johanna Czamanski-Cohen, Faisal Azaiza, Karen L. Weihs

**Affiliations:** ^1^The School of Creative Arts Therapies, Faculty of Social Welfare and Health Sciences, University of Haifa, Haifa, Israel; ^2^Emili Sagol Creative Arts Therapies Research Center, Faculty of Social Welfare and Health Sciences, University of Haifa, Haifa, Israel; ^3^Department of Psychiatry, College of Medicine, University of Arizona, Tucson, AZ, United States; ^4^The College of Sakhnin for Teacher Education, Sakhnin, Israel; ^5^Cancer Prevention and Control Program, University of Arizona Comprehensive Cancer Center, Tucson, AZ, United States

**Keywords:** breast cancer survivors, conservation, emotion processing, acceptance of emotions, psycho-oncology

## Abstract

**Introduction:**

Individuals from different cultures differ in their values, which encompass belief systems that individuals develop based on their culture, and play a pivotal role in shaping their perspectives. These values may affect emotion processing (EP): the recognition, interpretation, expression and response to bodily sensations, translated as emotions. These varying values may contribute to distinct emotional experiences, impacting physical and psychological symptoms in breast cancer (BC) survivors.

**Methods:**

This cross-sectional study investigated how EP including acceptance, expression (avoidance and approach coping), and awareness, may mediate the relationship between conservation values and symptoms of pain, fatigue, and depression among Arab (*n* = 62) and Jewish (*n* = 179) women BC survivors in Israel. Conservation values include tradition, conformity, and security.

**Results:**

Depression and fatigue were negatively correlated with acceptance of emotions, and positively correlated with avoidance and conservation levels. Emotion processing mediated the relationship between conservation and fatigue and depression. Arab women reported higher levels of various values, emotional acceptance, pain, fatigue, and depression symptoms compared to Jewish women. Conservation was higher in Arab women and correlated with both approach and avoidance coping which was not the case in Jewish women. Avoidance coping had a positive relationship with fatigue in the Jewish, but not the Arab women. Similarly, approach coping was negatively related to depression in Jewish, but not in Arab women.

**Discussion:**

Cultural differences are important for understanding the experience of cancer in individuals from different cultures. Future interventions for more conservative BC survivors should take culture into account.

## Introduction

Breast cancer (BC) is the most prevalent cancer in the world [World Health Organization (WHO), 2021]. It is a significant life event causing individuals to reevaluate their values and rebuild their identities. Values are important in determining how we respond to and cope with the challenges of a potentially life-threatening illness (Bedi and Devins, 2016; Mesquita et al., 2016). Individuals from traditional collectivistic cultural backgrounds often rely on religion as a major coping strategy, thus may have distinct reactions to cancer diagnosis and treatment. They tend to perceive their cancer diagnosis as predetermined fate, which often leads them to hesitate in expressing personal emotions and distress. This reluctance puts them at risk for experiencing feelings of loneliness and receiving inadequate support (Stanton et al., 2000a; Azaiza and Cohen, 2006; Cohen, 2014).

Israel is one of the world’s most ethnically diverse countries, with many multicultural signifiers, including nationality, ethnicity, and religion—emphasizing the latter (Halperin-Kaddari, 2000). The major ethnic diversity in Israel is between Jewish and Arab populations. The Jewish majority currently comprises 75% of the population, and the Palestinian-Arab Citizens of Israel (PACI) today make up about 21% of the population. Eighty-five percent of Arabs are Muslim, and the remainder are Christians, Druze, or other religions (Central Bureau of Statistics, 2022).

Palestinian-Arab Citizens of Israel experience lower levels of education, higher poverty rates, and less government support compared to Jews (Hammack, 2010; Daoud et al., 2018). Despite the Israeli healthcare system’s universal coverage, ensuring access equality for all citizens under the National Health Insurance Law (Mizrahi et al., 2009), there are significant socioeconomic disparities. Moreover, there are cultural differences between PACI and Israeli Jews. Palestinian-Arab Citizens of Israel are more collectivistic and traditional, while Israeli Jews are more Westernized and individualistic (Schwartz et al., 2012; Sortheix and Schwartz, 2017).

### Values

*Values* are belief systems that individuals develop based on the culture in which they are raised and live. At times, those values influence emotion processing (EP) as they have a role in defining the criteria by which a situation is judged and serve as guiding principles, mirroring individual self-perception and societal norms (Mesquita and Walker, 2003; Mesquita, 2010; De Leersnyder et al., 2015a; Schwartz, 2015; Sagiv et al., 2017). Schwartz’s theory Schwartz (1992) classifies values into 10 conflicting or compatible groups, with each group embodying a distinct set of principles and priorities. A later refinement by Schwartz (2017) identifies 19 specific values on a circular continuum. This updated theory also explores values’ relationships with gender, age, religiosity, and attitude. These values, organized in a circular structure, representing fundamental principles and proprieties, guide human behavior and decision-making. The 19 specific values are grouped into four higher-order categories: (1) Openness to change, includes values such as self-direction and stimulation, emphasizing independent thought and desire for novelty; (2) Self-Transcendence, which encompasses values like benevolence and universalism, prioritizing compassion and social justice; (3) Conservation, comprises values like tradition, conformity, and security, which emphasize respect for customs and the preservation of stability; (4) Self-Enhancement, includes values like power, achievement, and hedonism, focusing on the pursuit of success and personal gratification (Schwartz, 2016). These values form a comprehensive framework for understanding human behavior.

Israeli society presents a unique integration of these values orientations. It is a diverse and multicultural society, influenced by a rich tapestry of historical, religious, and cultural backgrounds (Halperin-Kaddari, 2000). There is a coexistence of traditional and modern values. While elements of collectivism are evident in strong emphasis on family and community ties, the nation also upholds individualistic principles (Schwartz et al., 2012). The presence of Arab women within the country adds an additional dimension of these dynamics (Dwairy and Jagelman, 2020). They are often part of traditional communities and may navigate a complex interplay of values. Simultaneously, the influences of modernization and westernization contribute to the adaption of more individualistic values, especially among younger generation including women (Dwairy, 2002; Abdo, 2011).

Achieving full equality between Arab and Jewish communities in Israel remains a challenge. This treatment may have fueled resilience among PACI, shaping their identity and influencing their demographic characteristics, such as level of education, employment, etc. (Dwairy and Jagelman, 2020). Perceptions of security, support, and conflict differ significantly between the two groups. As these groups interact, exploring the manifestation of the dynamics in Israeli society, especially regarding Arabs, is crucial for understanding cultural influences. This implies that within ethnically diverse urban settings, the identity of minority groups is less susceptible to erosion (Falah et al., 2000). Each culture endeavors to uphold its essential values.

Numerous studies have investigated personal values (Schwartz and Cieciuch, 2022). In recent years, psychological research has focused on personal values across cultures, examining their content, structure, and consequences. For example, Sagiv and Schwartz (1995) explored how personal values influenced readiness for outgroup social contact among Arab Israelis (*n* = 199) and Jews (*n* = 151). Aival-Naveh et al. (2022) developed a scale to measure mentalizing values (*N* = 360) and found that cultural differences in these values impacted thinking styles and emotional awareness. There is a prevailing understanding that cultural norms, practices, and values influence emotions and their processing.

### Emotions

Emotional states, influenced by past experiences and learned concepts, play a crucial role in our understanding of the world (Barrett, 2017). These states encompass the valence of emotional responses to internal and external situations, which, in turn, drive distinct behavioral reactions based on the perceived opportunities or challenges faced (Boiger et al., 2012; Gross, 2013). *Emotion processing* (EP) involves responding to the emotions generated by life experiences, allowing individuals to overcome intense feelings and navigate disturbances and distressing experiences (Baker et al., 2007). Higher EP through awareness, acceptance and expression of emotions, facilitates self-reflection and enhanced self-understanding (Rachman, 1980; Kanske and Kotz, 2011). A wide range of cultural differences in EP has been empirically demonstrated (Mesquita and Albert, 2007; Greenwood, 2012; Mesquita et al., 2016). *Emotional awareness* is a specific construct within emotional processing that involves recognizing and then describing emotions in oneself and others, signifying a cognitive skill that develops over time (Lane and Schwartz, 1987; Lane, 2006). Deficiencies in emotion awareness are linked to somatoform disorder (Subic-Wrana et al., 2010), and variances in emotional awareness indicate differences in how internal and external emotional information is processed, involving the differentiation and integration of related constructs (Chhatwal and Lane, 2016).

*Acceptance of Emotions* assesses how much individuals accept and nurture their emotions (Weihs et al., 2000). It is an important emotion regulation process involving the willingness to feel both positive and negative emotions and allow them to develop and dissipate without attempting to control, change, or reject them (Politi et al., 2007). Through actively engaging with nuanced and processed emotional experiences and embracing an accepting approach, individuals can enhance their ability to both express and effectively cope with their emotions (Pascual-Leone and Greenberg, 2007).

*Emotional expression* is an emotion-regulation strategy with salutary health effects and it has several social advantages including enhanced relationship satisfaction. Inhibiting emotion is deleterious to health, and the suppression of emotions is linked to adverse consequences such as reduced communication (Smyth, 1998; Butler et al., 2003; Sfeir et al., 2020). Emotional expression can provide information about internal states that are transformed in form and function into signals.

*Approach* and *Avoidance coping* represent distinct motivational mechanisms that contribute to emotional processing. Approach coping involves actively confronting emotional distress, encompassing acceptance and better management of the situation. Conversely, avoidance coping involves emotional, cognitive, and behavioral detachment from stressors (Bauer et al., 2016).

### Cultural differences in the experience of emotions

People from different cultures pay attention to different emotional cues (De Leersnyder et al., 2015b; de Gelder, and Huis in 't Veld, 2016). Analyzing emotion regulation within societies involves exploring cultural intricacies in shaping the occurrence, timing, and expression of emotions. Social values and norms establish unique emotion guidelines, deviating from the assumption of universally shared facial display. Local culture significantly influences individuals’ motivation and strategies in emotion regulation (Gross, 1998; John and Gross, 2007). Scholarly inquiry into the intricate relationship between cultural norms, practices, and values as they shape EP is sparse and additional investigation is needed to inform more appropriate interpretation and response to cultural differences in emotional experience.

Western culture is individualistic and encourages people to express their inner states (Lim, 2016), thus making positive and negative emotions predictors of life satisfaction, above the social norms (De Leersnyder et al., 2015b). By contrast, Easterners and individuals from collectivistic cultures hold values that emphasize interdependence, with the core unit of their society being the group, positing positive and negative emotions as less indicative of particular meanings, and predicting life satisfaction based on adherence to social norms (Suh et al., 1998; Von Scheve and Ismer, 2013; Lim, 2016). Hence, communal well-being is prioritized over individual benefit, aligning with the group’s (family or tribe) needs, rather than personal goals or feelings (Dwairy, 2002, 2004). Therefore, individuals from a collective and conservative background often restrain negative emotions due to their potential negative influence on familial and communal life (Mesquita, 2001; Seyfert, 2012). Socialization further reinforces the inhibition of negative emotions that might disrupt family and group objectives. This distinct honor culture significantly influences emotion management, diverging from the dignity norm in Western countries (Levin et al., 2015).

These societies shape collective personalities, social norms, values, roles, authority, and coping skills (Dwairy, 2002). This tendency sharply contrasts with Western society, which stresses individualism, self-actualization, and pursuing personal goals. This dissimilarity surfaces in the interpretation of emotions. For example, the Western perspective of depression is predominantly characterized by sadness and anhedonia, whereas collective societies often report it with more somatic complaints and fewer mood changes. Thus, the same emotion is valued—and might be experienced—differently and has different consequences for relationships and behavior (Dwairy, 2004; Boiger et al., 2013, 2018).

This aligns with traditional Arab culture, which tends to reinforce the non-expression of negative emotions, justified by the high value that Arab society places on preserving harmony within the family and the need to prioritize this value at the expense of personal needs and goals (Dwairy, 2002). Yet, to the best of our knowledge, EP within Arab and collective societies remains underexplored, particularly concerning life-threatening events like BC.

### Ethnocultural contexts of emotions and coping with Cancer

In western society, emotions play a significant role throughout cancer experiences, from diagnosis to treatment and beyond (Halldórsdóttir and Hamrin, 1996; Traeger et al., 2013; Wirkner et al., 2017; Martino et al., 2019; Ogińska-Bulik and Michalska, 2020). Modifying emotional processes can improve patient health outcomes, with increased awareness and acceptance of emotions linked to better psychological and physical well-being (Ferrer et al., 2015; Czamanski-Cohen and Weihs, 2016). Acceptance of emotions is associated with lower depression (Stanton et al., 2018) and improved health and quality of life for BC patients, benefiting both younger and older women (Brandão et al., 2016; Marroquín et al., 2016; Reed et al., 2016). Furthermore, higher emotional acceptance predicts decreased mortality even after accounting for emotional distress (Politi et al., 2007; Weihs et al., 2008). Although the cancer experience may evoke intense emotions during active treatment and long after, individuals differ substantially in their tendency to express emotion, which is affected by personal developmental processes and ethnicity and religiosity (Gross, 2013).

Cancer survivorship, defined as living with the challenges of cancer diagnosis and treatment, has significant emotional and social implications (Cohen, 2014; Goldblatt et al., 2016; Granek et al., 2019; Abu-Odah et al., 2022). Furthermore, BC, a disease predominantly affecting women, presents distinctive challenges in terms of their psychological well-being. Established gender roles and societal expectations often position women as primary caretakers, and as such, they are more susceptible to encountering stressful events that can have adverse consequences on their overall health and self-care practices (Ayala et al., 2017; Almegewly and Alsoraihi, 2022). Furthermore, the experience of being a woman from a minority group in a predominantly Western-centric environment may introduce intricacies and nuanced challenges throughout their journey (Culver et al., 2004; Abu Farha et al., 2017; Abdel-Salam et al., 2019; Awad et al., 2022).

In studies with female BC survivors holding more traditional values, participants’ expression of positive emotions are often related to their strong belief in God as a source of support and recovery. This faith, that may be reinforced following diagnosis, can induce women to believe they will be granted a cure. In a prospective study with Arab BC survivors, those who identified as Muslim exhibited a pronounced acceptance of their fate in comparison to survivors who were Christian (Goldblatt et al., 2016). However, women from ethnocultural and faith-based communities often face barriers to death behaviors and early screening due to beliefs associating cancer with causation or slow development (Freund et al., 2019). These communities’ social and religious beliefs play a dual role in shaping women’s reactions to the disease, offering both sources of strength and challenges (Bedi and Devins, 2016). Extensive research has shown that in collective societies, such as Arab communities, women may perceive cancer as fate and fear societal stigma, leading to restrained emotional expression and delayed medical attention (Stanton et al., 2000a; Low et al., 2006; Cohen, 2014). These cultural dynamics are evident in Arab women living in Israel, who exhibit lower BC screening rates and express concerns about societal roles, leading to unexpressed distress and poorer well-being. Arab women prioritize their mothering roles within the collective society (Abdo, 2011). Consequently, they harbor concerns that a BC diagnosis might disrupt their established roles as both wives and mothers (Azaiza and Cohen, 2006, 2008; Cohen and Azaiza, 2008; Goldblatt et al., 2016).

Breast cancer patients frequently use coping strategies including social support, positive reframing, religious efforts, emotional expression, and avoidance/distraction (Assaf, 2011). Coping strategies vary by ethnicity, education, and disease phase (Khawaja, 2008; Mehrabi et al., 2015). In a quantitative study of Muslim women with BC (*N* = 62), coping strategies included religion, acceptance, avoidance, and denial. Denial and avoidance were common and were related to more physical symptoms, pain. They also had negative impacts on relationships and enjoyment of life, ultimately affecting overall quality of life (Khalili et al., 2013). A qualitative study of Arab BC survivors (*N* = 19) revealed that Islamic beliefs were the most common coping strategy, accompanied by denial, optimism, withdrawal, and family/healthcare support. Optimistic women using positive cognitive strategies experienced better psychological health than those using avoidance (Al-Azri et al., 2014). However, isolation and prayer were found as sources of solace in another qualitative study (*N* = 20) for Arab BC survivors (Assaf et al., 2017).

Given the above-mentioned views of the experience of cancer in Arab societies, which emphasize the acceptance of fate, and the tendency of eastern societies to experience negative emotions somatically, it is of interest to deepen our understanding of how acceptance of emotions and their expression and coping strategies are related to the experience of symptoms in Arab BC survivors, and to further understand the relationship between their emotions and well-being. In this study, we examine how culture and personal values relate to individual emotion processing (EP), physical experience (pain and fatigue), and psychological (depression) symptoms in BC survivors from collectivistic and individualistic cultural backgrounds among Arabs and Jews in Israel. Thus, the current study aims to address a gap in the literature by examining if Arab BC survivors have higher levels of acceptance of emotion and lower expression, and if EP mediates the relationship between conservation values and depression, pain and fatigue.

### Research hypotheses

We hypothesize that:

(*H1*) Conservation will be positively related to acceptance of emotion and avoidance coping and negatively related to approach coping and emotional awareness.

(*H2*) Arab BC survivors will report higher conservation, acceptance of emotion and avoidance coping and lower approach coping than Jewish BC survivors.

(*H3*) EP will mediate the relationship between conservation and depression, pain, and fatigue.

(Exploratory hypothesis) EP will mediate the relationships between conservation values and symptoms among Arabs and Jews, differently.

## Methods

To obtain our study aims, we conducted a cross-sectional quantitative study to examine the relationships between values, emotional processing, depressive symptoms, pain, and fatigue among Israeli Jewish and Arab women who recently completed initial BC treatment. The primary outcome was EP, comprising emotion awareness, expression (avoidance and approach coping), and acceptance; secondary measures were depression, pain, and fatigue. This study was part of a larger research effort examining EP’s role as a mechanism of symptom reduction through art therapy in Arab and Jewish Israeli BC survivors. Our research was conducted on the baseline data of this randomized controlled trial (Czamanski-Cohen et al., 2020).

### Procedure

Participants were recruited from several hospitals and community sites in Israel between May 2019 and March 2022. The “*REPAT*” study team approached participants, emphasizing the importance of addressing each participant in their language. Once eligibility was established, participants signed informed consent and then completed baseline questionnaires in Arabic or Hebrew, online or on paper. The anonymous patient-reported data were collected and managed using REDCap electronic data capture tools hosted at the University of Arizona (Harris et al., 2019).

### Participants

Inclusion criteria were adult (18 years or older) Israeli Jewish or Arab women with initial, first-time or recurrent BC or second primary BC; three months after finishing chemotherapy and radiotherapy or one month post-surgery (whatever occurs last) and less than 12 months after the end of primary chemotherapy and radiotherapy or surgery. Additional or replacement standard medical treatment for cancer was allowed. Participants could complete assessments in Arabic or Hebrew; and provided informed consent. Exclusion criteria were men; women with a lifetime history of bipolar disorder, schizophrenia, schizoaffective disorder, or a precancer fibromyalgia or chronic fatigue syndrome diagnosis; an active suicide plan (we ensured immediate intervention); dementia or other disorders precluding informed consent or assessment comprehension; taking anticholinergic medications, post myocardial infarction (6 months before recruitment), or pacemaker; or flare-up in systemic autoimmune disease or thyroid dysfunction requiring increased medication. The final sample was comprised of 179 Jewish and 62 Arab women.

### Measures

#### Baseline demographic and ethnocultural differences

We collected baseline *demographic data* including age, marital status, children (if yes, how many), religion, religiosity, education, employment, residence (city, village, etc.). *Values* were measured with the Portrait Values Questionnaire, a 57-item scale measuring 19 cultural values, to measure ethnocultural differences. The questionnaire has been validated in 15 samples across 10 countries (*N* = 6,059), including Israel (Schwartz et al., 2012). The mean Cronbach’s alpha for tradition values (used as a covariate to examine ethnocultural differences between Jews and Arabs) was 0.83. The Portrait Values Questionnaire value scale was validated and translated by the author, Schwartz, into numerous languages with the use of accepted translation methods (Schwartz et al., 2001).

#### Emotion processing

##### Emotional awareness

We measured emotional awareness with the 10-item Levels of Emotional Awareness Scale (LEAS) which is a written performance index of the ability to express emotion in different and complex ways (Lane et al., 1990). Subjects write their and another person’s anticipated feelings in response to short vignettes. Responses are scored from 0 to 5 on the range of emotions described and the terms’ specificity. We translated the LEAS from English to Hebrew and Arabic and back-translated to English and completed a validation study of the scale in Hebrew and Arabic (*N* = 130, Hebi et al., 2020). The total scores’ interrater reliability between two independent raters was above 0.81 (Lane et al., 1990).

##### Emotional expression

We assessed emotional expression by the Situational Avoidance and Approach Coping scales (Bauer et al., 2016). The Situational *Avoidance* Coping scale is a 12-item scale with three subscales from the COPE scale (denial, behavioral disengagement and mental disengagement); the Situational *Approach* Coping scale is a 24-item scale with four subscales from the COPE scale (Carver et al., 1989) (acceptance, positive reinterpretation, problem-solving and social support), and 8 items from the emotional expression and emotional processing subscales of Coping through Emotional Approach scale (Stanton et al., 2000b). The Situation Avoidance and Approach Coping scales (Bauer et al., 2016) were translated for the present study from English into Arabic and Hebrew by two bilingual experts using the back translation method and tested in a pretest phase (not published yet).

##### Acceptance of emotion

We assessed emotional acceptance with the Acceptance of Emotion Scale, a 13-item self-report that asks participants to rate (10–100) how similar they are to a described individual relative to the extent they accept and nurture their feelings. It had been validated in a study of 160 BC patients (α = 0.92; Weihs et al., 2008). This scale was translated from English to Hebrew and Arabic and back-translated to English.

#### Psychological symptom: depression

Depression symptoms were measured using the 10-item Center for Epidemiologic Studies-Depression scale (CES-D; Radloff, 1977), on which participants rated how often they experienced depressive symptoms. The CES-D is a widely used dimensional measure of depression with good psychometric properties in community and BC-diagnosed populations. The scale’s reliability and validity have been tested in general and clinical populations, yielding very good internal consistency (α = 0.85 for general and α = 0.90 for psychiatric populations; Hann et al., 1999). This scale was validated in Arabic (*N* = 435) in a community sample of Lebanese adults (Kazarian and Taher, 2010). It was also translated into Hebrew using the back-translation method (Levkovich et al., 2015).

#### Physical symptoms

##### Pain

We used six pain items from the PROMIS Pain Intensity Scale and three from the Pain Impact Scale (Cella et al., 2010) to measure how much and how intensely pain interfered with different aspects of the participants’ lives the past 4 weeks. Reliability was assessed as internal item consistency for each PROMIS measure. Generally, a Cronbach’s alpha above 0.70 is acceptable for both reliability types (Deyo et al., 2015). Cella et al. (2010) found α > 0.90. The scales were translated from English to Hebrew and Arabic using the back-translation method (Eremenco et al., 2005).

##### Fatigue

We assessed participants’ fatigue using the Fatigue Symptom Inventory (Hann et al., 2000). This 14-item self-report was developed and validated specifically among BC patients/survivors. It demonstrated high internal consistency, yielding internal reliability (α > 0.90) in a previous study conducted in Israel. That study also translated the questionnaire from English into Hebrew by two bilingual experts using the back-translation method and tested it in a pretest phase (Cohen and Fried, 2007; Cohen et al., 2012), with α ≥ 0.91 (Cohen and Fried, 2007), and Arabic α = 0.89 (Levkovich et al., 2015).

All questionnaires are available in both Arabic and Hebrew versions, and these versions share an identical factorial structure.

### Data analysis

Using IBM SPSS (Version 27), we conducted statistical analyses to examine the study hypotheses. We calculated descriptive statistics for all variables using means and standard deviations. Pearson correlations were used to assess correlations between the study variables (H1). We used Independent Samples t- tests to examine the means differences in values, EP indices, and psychological and physical symptoms in Jewish and Arab BC survivors (H2).

To test the mediation model Structural Equation Modeling (SEM) using MPLUS7 (Muthén and Muthén, 1998–2007) was applied. Missing data were completed using maximum likelihood estimation under MCAR (missing completely at random) and MAR (missing at random; Little and Rubin, 2002). All variables in the model are observed ones. Conservation was used as a predictor variable. A regression model was estimated to test whether the conservation level predicts symptoms of depression, pain, and fatigue through the mediating variables of EP: Acceptance of Emotions, Levels of The Emotional Awareness Scale (LEAS), and Situational Avoidance and Approach Coping scales (H3). To test the differences between Arabs and Jews, A multigroup structural equation modeling approach was used to compare the model between the two groups (Exploratory hypothesis).

## Results

### Demographics

Of the 241 study participants, 39% had stage 2 BC; 179 (74%) were Jewish, 62 (26%) were Arab, 68% were recruited from the community, and 32% from hospitals. In age, 6% were 26 to 35; 21% were 36 to 45; 66% were 46 to 70 years; and 7% were 70 years or older; and 28% held an undergraduate degree (32% Jews, 15% Arabs).

50% were employed (58% Jews, 29% Arabs), and 57% had average incomes (50% Jews, 79% Arabs). The Arab sample comprised 62% Muslims, 32% Druzes, and 3% Christians. Most of the Arab women considered themselves religious (52%); 55% of the Jewish women considered themselves secular.

Table 1 shows descriptive statistics.

**Table 1:** Descriptive statistics.

Measure		Total *N* = 241		Hebrew *N* = 179		Arabic *N* = 62		Hebrew	Arabic	*t-*test
		*n*	%	*n*	%	*n*	%	*M* (*SD*)		
Religion	Jewish Muslim Christian Druze Other	176 39 2 20 2	73.6 16.3 0.8 8.3 0.8	176 - - - 2	99.4 - - - 1.1	- 39 2 20 -	- 62.9 3.2 32.2 -			
Age group	26–35 36–45 46–70 Over 70	12 52 159 18	5.0 21.2 66.2 7.5	6 35 121 17	3.4 19.6 67.4 9.5	6 17 38 1	9.7 27.4 61.3 1.6	2.8 (0.63)	2.50 (0.69)	3.15 (239)**
Religiosity	Secular Religious Traditional	144 63 64	60.2 26.3 26.5	99 31 49	55.3 17.3 27.4	15 32 15	24.2 51.6 24.2	1.7 (0.86)	2.00 (0.70)	2.31 (239)*
Education	High school Certificate Undergraduate Graduate school Other	59 40 67 60 12	24.7 16.8 28.1 25.2 5.0	28 34 58 52 4	15.9 19.3 32.9 29.5 2.2	31 6 9 8 8	50 9.6 14.5 12.9 12.9	1.9 (0.81)	1.30 (0.68)	4.61 (239)**
Income	Below average Average Above average	32 137 69	13.4 57.5 28.9	24 88 64	13.6 50.0 36.3	8 49 5	12.9 79.0 8.0	2.2 (0.67)	1.90 (0.45)	3.05 (237)**
Marital status	Single Married Divorced Widowed Other	20 177 33 9 2	8.2 73.4 13.6 3.7 0.8	16 126 26 9 1	8.9 70.7 14.6 5.0 0.5	4 51 7 - 1	6.4 82.2 11.2 - 1.6	2.1 (0.67)	2.05 (0.42)	1.32 (239)**
Cancer stage	0 1 2 3 4	7 73 89 46 13	3.0 32.0 39.0 20.1 5.7	4 58 72 30 8	2.3 33.7 41.8 17.4 4.6	3 15 17 16 5	5.3 26.7 30.3 28.5 8.9	1.8 (0.89)	2.10 (1.03)	1.89 (233)*
Depression								10.5 (5.80)	12.80 (6.90)	2.55 (238)*
Fatigue								57.6 (28.80)	74.30 (26.00)	4.02 (238)**
Pain intensity								55.8 (12.60)	64.00 (9.60)	4.60 (238)**
Emotion processing	Emotional expression	Approach Avoidance						2.3 (0.67) 1.8 (0.47)	2.70 (0.66) 2.40 (0.57)	3.90 (217)** 7.90 (223)**
	Emotional acceptance							63.5 (23.30)	70.40 (20.50)	2.07 (239)*
	Emotional awareness							28.4 (6.40)	30.10 (6.10)	1.80 (239)
Personal value	Conservation							4.3 (0.90)	4.90 (0.70)	4.70 (227)**

*p ≤ 0.05, **p ≤ 0.005.

### Correlations between demographics and study variables

Results showed positive correlation between education, income (*r* = 0.42**, *p* < 0.01), and approach (*r* = 0.17*, *p* < 0.05). There were negative correlations between education and religiosity (*r* = −0.18**, *p* < 0.01), fatigue (*r* = −0.19**, *p* < 0.01), pain intensity (*r* = −0.30**, *p* < 0.01), avoidance (*r* = −0.21**, *p* < 0.01), and conservation (*r* = −0.13*, *p* < 0.05).

Income correlated negatively with depression (*r* = −0.25**, *p* < 0.01), fatigue (*r* = −0.29**, *p* < 0.01), pain intensity (*r* = −0.21**, *p* < 0.01), and conservation (*r* = −0.16*, *p* < 0.05), and correlated positively with approach (*r* = 0.14*, *p* < 0.05).

### Correlations between personal values, emotion processing, and symptoms

Results showed positive correlation between conservation and pain intensity (*r* = 0.24***, *p* < 0.001), avoidance (*r* = 0.21***, *p* < 0.001), approach (*r* = 0.27***, *p* < 0.001), and acceptance of emotions (*r* = 0.31***, *p* < 0.001). Results did not show significant correlations between conservation and emotional awareness, fatigue, or depression (H1). Depression correlated positively with fatigue (*r* = 0.49***, *p* < 0.001), pain intensity (*r* = 0.27***, *p* < 0.001), and avoidance (*r* = 0.24***, *p* < 0.001). Depression correlated negatively with acceptance of emotions (*r* = −0.43***, *p* < 0.001). Fatigue correlated positively with pain intensity (*r* = 0.45***, *p* < 0.001), and avoidance (*r* = 0.30***, *p* < 0.001), and negatively with acceptance of emotions (*r* = −0.21**, *p* < 0.01). Also, positive correlations between approach and acceptance of emotions (*r* = 0.46***, *p* < 0.001), acceptance of emotions and emotional awareness (*r* = 0.15*, *p* < 0.05) were reported. Table 2 shows correlations for the study variables.

**Table 2:** Correlations of study variables.

			Personal values	Emotion processing				Physical symptoms	
			Conservation	Expression		Acceptance	Awareness	Pain	Fatigue
				Avoidance	Approach				
Emotion Processing	Expression	Avoidance	0.211***						
		Approach	0.274***	0.030					
	Acceptance		0.311***	0.054	0.462***				
	Awareness		0.092	0.038	0.080	0.151*			
Physical symptoms	Pain		0.248***	0.127	−0.043	−0.078	−0.086		
	Fatigue		0.099	0.303***	0.023	−0.215**	0.080	0.453***	
Psychological symptoms	Depression		0.042	0.240***	0.107	−0.432***	0.093	0.276***	0.493***

*p ≤ 0.05; **p ≤ 0.01; ***p ≤ 0.001.

### Differences between Arab and Jews

Regarding cancer stage, Arab women (*M* = 2.10, *SD* = 2.03) had a higher prevalence of advanced stages compared to Jews (*M* = 1.8, *SD* = 0.8), *t*(233) = 1.8, *p* < 0.05. Out of the total women diagnosed with stage 3, Arabs had a higher incidence (28.5%) compared to their overall representation (20.1%), while Jewish women had a lower incidence of this stage (17.4%). Arab women also had a higher incidence of stage 4 cancer (8.9%) compared to their overall representation (5.7%), whereas Jewish women had a lower incidence of stage 4 cancer (4.6%) compared to their overall representation.

Arab participants reported higher scores on conservation, which consists of conformity (interpersonal and rules), security (personal and societal), and tradition(*M* = 4.9, *SD* = 0.7) than Jews (*M* = 4.3, *SD* = 0.9), *t*(227) = 4.7, *p* < 0.001. Arab participants reported higher avoidance coping levels (*M* = 2.4, *SD* = 0.57) than the Jewish participants (*M* = 1.8, *SD* = 0.47), *t*(223) = 7.98, *p* < 0.001. As hypothesized, acceptance of emotions was higher among the Arab (*M* = 70.4, *SD* = 20.5) than the Jewish (*M* = 63.5, *SD* = 23.3), *t*(239) = 2.07, *p* < 0.05 participants. However, the Arabs reported higher depression levels (*M* = 12.8, *SD* = 6.9) than the Jewish participants (*M* = 10.5, *SD* = 5.8), *t*(238) = 2.55, *p* < 0.05); higher fatigue levels (*M* = 74.3, *SD* = 26.0) than Jews (*M* = 57.6, *SD* = 28.8), *t*(238) = 4.02, *p* < 0.001; and higher pain intensity (*M* = 64.0, *SD* = 9.6) than Jews (*M* = 55.8, *SD* = 12.6), *t*(238) = 4.6, *p* < 0.001 (H2).

Table 3 shows the correlation analysis results for the study variables within both the Arab and Jewish participant groups.

**Table 3:** Correlations of study variables for Jews versus Arabs.

			Personal values	Emotion processing				Physical symptoms	
			Conservation	Expression		Acceptance	Awareness	Pain	Fatigue
Jews (*N* = 179)				Avoidance	Approach				
Emotion Processing	Expression	Avoidance	−0.015						
		Approach	0.141	0.074					
	Acceptance		0.264***	−0.047	0.501***				
	Awareness		0.098	0.074	0.131	0.168*			
Physical symptoms	Pain		0.132	0.025	−0.039	−0.114	−0.079		
	Fatigue		0.043	0.304***	−0.021	−0.174*	0.115	0.403***	
Psychological symptoms	Depression		−0.032	0.263**	−0.022	−0.366***	0.055	0.248***	0.495***
Arabs (*N* = 62)									
Emotion Processing	Expression	Avoidance	0.302*						
		Approach	0.672***	0.210					
	Acceptance		0.364**	0.108	0.570***				
	Awareness		−0.037	−0.150	0.037	0.035			
Physical symptoms	Pain		0.280*	0.173	0.025	−0.078	−0.191		
	Fatigue		0.011	0.137	0.021	−0.132	−0.130	0.541***	
Psychological symptoms	Depression		−0.164	0.112	−0.214	−0.371**	0.019	0.330**	0.702***

*p ≤ 0.05; **p ≤ 0.01; ***p ≤ 0.001.

### The mediation model

**Figure 1:**
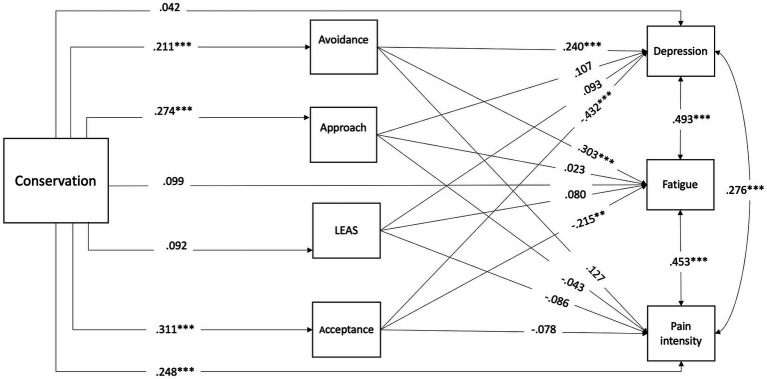
The final estimated model based on the structural equation modeling analysis **p ≤ 0.01; ***p ≤ 0.001.

Overall, the model provided a good fit with the data according to fit criteria suggested by Hu and Bentler (1999) (*χ*^2^/df = 1.11, *p* = 0.35, CFI = 0.99, TLI = 0.98, SRMR = 0.03, and RMSEA = 0.02) and is presented in Figure 1, which includes standardized estimates of the parameters in the structural model. Higher avoidance mediated the effect of conservation on fatigue and depression. Also, higher acceptance mediated the effect of conservation on fatigue and depression.

Our findings support the hypothesis that EP is involved in mediating depression, pain, and fatigue symptoms. Estimations of this model showed that conservation was positively associated with avoidance which mediated the impact of conservation on higher levels of both fatigue and depression. Similarly, conservation was positively associated with acceptance, which predicted lower levels of both fatigue and depression. In addition, conservation was positively associated with approach, but approach did not significantly predict depression, fatigue, or pain. The total path between conservation levels and pain was significant and none of the mediation variables mediated this relationship (H3).

A multigroup structural equation modeling approach was used to compare the model between Jews and Arabs. To test for weak factorial invariance (Meredith, 1993) across groups, the chi-square from a model with all parameters allowed to be unequal across groups was compared to the chi-square from a model with only the loadings constrained to be equal across groups. No means or intercepts were estimated in these models.

The model with all parameters freely estimated in the two groups, fit the data very well, CFI = 0.99, SRMR = 0.04, *χ*^2^(10) = 9.00 *p* = 0.53 according to fit criteria suggested by Hu and Bentler (1999). The weak invariance model with loadings constrained to be equal across groups had fit that was significantly poorer, *χ*^2^(30) = 53.05, *p* < 0.01, ∆*χ*^2^(20) = 44.06, *p* < 0.01. The Comparative Fit Index for this model indicated good fit but poorer than the unconstrained model, CFI = 0.92, the SRMR was 0.09, which suggested the fit could be improved.

Examining path by path differences estimations for Jews versus Arabs revealed four significant differences (see Table 4): 1. Conservation has a higher positive relationship with approach among the Arabs than among the Jews (β = 0.66 vs. β = 0.14, *p* < 0.001). 2. Conservation has a positive relationship with avoidance among the Arabs and has no relationship with avoidance among the Jews (β = 0.30 vs. β = 0.00, *p* = 0.03). 3. Avoidance has no relationship with fatigue among the Arabs and has a positive relationship with fatigue among the Jews (β = 0.04 vs. β = 0.27, *p* = 0.045). 4. Approach has no relationship with depression among the Arabs and has positive relationship with depression among the Jews (β = 0.05 vs. β = 0.18, *p* = 0.049) (H4).

**Table 4:** Multigroup analysis of paths for Jews versus Arabs when different.

	*χ* ^2^	df	Δ*χ*^2^ from the base model	Estimate Jew	Estimate Arab
Constrained base model^a^	53.01	30			
Unconstrained path^b^					
Conservation → Approach			17.4***	0.14	0.66***
Conservation → Avoidance			4.42*	0	0.30*
Avoidance → Fatigue			4.00*	0.27***	0.043
Approach → Depression			3.85*	0.16*	0.05

a Paths for the two groups were allowed to be equally estimated.

b The path specified was NOT constrained to be equal across the two groups.

*p ≤ 0.05; **p ≤ 0.01; ***p ≤ 0.001.

## Discussion

Research indicates that BC survivors contend with diminished quality of life (Marroquín et al., 2016), mirroring elevated levels of depression, pain, and fatigue conveyed by our participants. Notably, significant correlations were identified among avoidance coping, depression, fatigue, and pain, underscoring the intricate relationship between mental well-being and quality of life (Reed et al., 2016). Additionally, the notion that EP contributes to symptom management has been proposed (Ogińska-Bulik and Michalska, 2020). This concept aligns with the observed negative correlation between acceptance of emotions and depression, as well as fatigue: Women who exhibited greater acceptance of emotions tend to experience lower levels of depression and fatigue, reinforcing the existing literature on EP and its impact on health outcomes, leading to enhanced psychological and physical well-being (Politi et al., 2007; Ferrer et al., 2015; Brandão et al., 2016; Czamanski-Cohen and Weihs, 2016; Stanton et al., 2018).

Regardless of the collectivist culture’s impact on BC, the relationship between conservation and avoidance remains consistent, especially in situations where discussing personal experience is challenging (Azaiza and Cohen, 2006; Abdo, 2011). This aligns with prior research, which has shown that denial and avoidance tend to be prevalent coping strategies among BC survivors with collectivist values (Khalili et al., 2013; Al-Azri et al., 2014). These strategies have been associated with more adverse physical symptoms, including pain and lower quality of life (Khalili et al., 2013). Our findings further support this connection, as we report a positive correlation between avoidance and both fatigue and depression. In addition, we found a positive correlation between conservation and approach. Since a conservative person avoiding something might not simultaneously employ an approach coping strategy, it may be the case that the approach coping is being endorsed due to its associations with connecting with others and sharing personal experiences to cope positively with cancer. However, this connection appears ambiguous and potentially contradictory. If these women associate their conservation level with avoidance, it seems incongruous for them to also employ approach coping strategies. Overall, we observed that Arab women reported elevated levels of conservation values, EP, and symptoms compared to Jews. This pattern could be linked to the tendency observed in studies involving participants from collectivist cultures to provide the “correct” response or exhibit politeness consistently (Matsumoto et al., 2008) which in this study may be demonstrated in the seemingly contradictory associations of both acceptance and avoidance of emotions with higher reported levels of conservation.

The positive association of conservation with acceptance of emotions, which consequently negatively predicted fatigue and depression has not previously been reported, to our knowledge. Considering the strong relationship between conservation and religiosity as highlighted in our results, we can draw insights from existing literature. Several studies on cancer indicate that the perception of God’s image can influence coping and psychospiritual well-being among patients (Schreiber, 2011). Additionally, faith in God often offers solace, purpose, and resilience, and it has been noted to rise in women who perceive a deep involvement of God (Schreiber, 2011; Goldblatt et al., 2013). Given Arab culture’s collectivist and conservative nature, we hypothesized and confirmed that Arab participants report higher acceptance of emotions levels. This suggests that religious individuals with strong conservation values may adopt coping strategies influenced by their collectivist and conservative culture. Similarly, in previous studies among Arab Muslim BC survivors, Islamic spiritual practices were documented to be the most common coping strategy, prayer was found as a source of solace, and those who adopted positive cognitive strategies experiences better psychological health compared to those who relied on avoidance (Al-Azri et al., 2014; Assaf et al., 2017). This interpretation implies that collectivism and religiosity could potentially offer positive coping strategies within these groups, stemming from the deeply integrated cultural values, which emphasis harmony, resilience without complaint, and trust God’s will (Dwairy, 2002; Seyfert, 2012). It may be these beliefs form the foundation for the coping strategies for Arab Muslim BC survivors.

In conducting a more comprehensive analysis of our results, it is imperative to underscore the significance of sociodemographic factors, notably the observed low educational attainment among PACI women. Our findings highlight health disparities, emphasizing the necessity of exploring underlying determinants. The documented socioeconomic challenges, including lower education level among PACI may contribute to the observed health contrasts. However, the study revealed that Arab women were diagnosed with more advanced BC stages and severe conditions. This may be linked to factors like insufficient self-care practices influenced by cultural norms, as well as the role of women within the society. Additionally, discrimination and stressors prevalent among minority groups, including lower socioeconomic status and limited educational access (Daoud et al., 2018) may contribute. Moreover, the Arabs reported higher depression levels than the Jewish participants; higher fatigue levels; and higher pain intensity than Jews. Questions might arise about the relationship between their low quality of life (as indicated by their symptoms) and high acceptance of emotion levels, whether social harmony and religious priorities influence their perceptions of acceptance, and how they deal with such life-threatening situations. Moreover, the positive influence of conservation associated with avoidance, subsequently predicting higher fatigue and depression, prompts further exploration of factors related to conservation’s tie to low quality of life. This phenomenon aligns with traits of collectivist societies, which prioritize group bonds and communication within the collective over the needs of individuals (Dwairy, 2002; Seyfert, 2012; Lim, 2016). Among Arabs, this is exemplified by conservation’s positive influence on avoidance, unlike Jews, most of whom identify as secular. This consideration is crucial for understanding why the total path between conservation levels and pain was significant and none of EP variables mediated this relationship. Likely, due to cancer’s negative connotations, some women might view illness as a manifestation of God’s punishment or will (Azaiza and Cohen, 2006). Social and religious beliefs play dual roles—sources of strength and frustration—in Arab BC survivors’ reactions to their disease in the personal, familial, and societal spheres (Goldblatt et al., 2016).

Studies have highlighted cultural contexts as potential drivers of individual differences in EP (Mesquita, 2001; De Leersnyder et al., 2015a; Boiger et al., 2018). Cultural differences should continue to be recognized as potential sources of variation in needs and experience. For instance, we did not find the expected positive relationship between avoidance and fatigue, nor a negative relationship between approach and depression in Arab participants. While our study illuminates Arab women’s values, EP and symptoms, and the relationships between them, it still remains unclear how Arab women cope and process their emotions during the cancer experience. This calls for further, possibly qualitative, research to obtain contextualized data to deepen our initial results, particularly within the realm of BC.

Understanding the impact of cancer’s emotional journey, influenced by individual development and ethnic backgrounds (Mesquita and Albert, 2007; Mesquita et al., 2016), is crucial for effective psychosocial interventions, especially in traditional communities. Tailored interventions should comprehensively address cultural nuances embedded in collectivist values. These interventions would benefit from considering the educational and socioeconomic disparities prevalent in this population, as these factors significantly impact their overall quality of life.

The current study aimed to examine whether EP mediates the relationship between conservation and cancer related sickness symptoms in order to deepen our understanding of how cultural norms and emotional experience may interact and affect cancer survivors’ quality of life. This information can be used in the development of effective psychosocial interventions and could serve as a bridge between health care providers and patients in traditional communities. We advocate for healthcare professionals, particularly those in oncology, to support patients’ coping strategies and foster authentic emotional processing that aligns with both their individual and societal perspectives. Additionally, it is vital to offer guidance to family and friends about their role in the process.

### Importance and innovation of research

Besides examining how personal value perceptions in coping with BC might be culturally different, this study offers several contributions to the field of ethnocultural differences in EP. However, a comprehensive study of the cultural aspects of cancer in ethnic and cultural groups worldwide is necessary to deepen our understanding of cancer care needs, especially among collectivist and minority groups. Our results shed some light on how culture influences emotional, psychological, and physical symptoms. Culturally competent and sensitive interventions for BC survivors may improve their influence on symptoms. This study expands our understanding of the Israeli and Arab populations, which the literature has not widely addressed, especially in terms of EP. Our findings demonstrate that Arab women have significantly higher levels of depression, pain, and fatigue, which they may not independently report to caregivers. Thus, initiating care during BC treatment, and providing culturally appropriate interventions, may help reduce this disparity. This article calls for developing appropriate therapeutic interventions for EP and coping with cancer that may help patients better manage and cope with psychological and physical symptoms.

## Limitations

The study faced challenges due to imbalanced participant distribution, with fewer Arab participants than Jewish. Recruiting Arab participants was also impeded by cultural factors and limited accessibility. Additionally, the Covid-19 pandemic’s impact on data collection may have influenced participation and study dynamics. Lastly, depending solely on baseline data could pose a limitation, as adopting only a quantitative approach might constrain the depth of comprehension.

### Future directions

An investigation into the positive correlation between conservation and approach, that remains unexplained, is essential. Additionally, our findings demonstrate differences in symptom experiences between Arab and Jewish women. Future studies should investigate coping strategies and EP specific to Arab BC survivors to gain a deeper understanding of these patterns. Also, conducting an in-depth exploration of traditional and religious Arab and Jewish women, along with a comparative analysis, stands to significantly enhance our understanding and knowledge. Lastly, we recommend a medium other than verbal to better understand EP and how it can be expressed. Because reporting a life-threatening experience like cancer is not easy for everyone, especially individuals with little experience expressing inner feelings. In another part of the larger study, we examine EP (expression) through an art therapy intervention to understand better how nonverbal expression relates to the participants’ scores.

## Conclusion

Our study highlights the role of EP among communities coping with BC holding prioritized personal values such as conservation, and how their EP was related to depression, pain, and fatigue symptoms. Our findings showed that higher EP is associated with lower depression and fatigue, and consistent with previous studies, avoidance was associated with higher levels of fatigue and depression among BC survivors. These findings align with existing research on EP’s benefits to well-being. Our findings indicate a positive correlation between conservation and approach, though the reason for this is unclear.

Between the two groups, the Arab women were significantly more likely to experience sickness symptoms than the Jewish women. Moreover, they reported higher levels of conservation and EP than Jewish women, possibly due to cultural tendencies toward socially desirable responses. This suggests the need for further investigation into how cultural differences influence responses to written questions about emotion processing in cancer patients. Only a few studies have examined how Arab women cope with BC or how the illness affects their lives. This study may help health care providers enhance care quality and efficiency for women by integrating their core cultural values into clinical practice.

## Data availability statement

The original contributions presented in the study are included in the article/supplementary material, further inquiries can be directed to the corresponding author.

## Ethics statement

The studies involving humans were approved by the Ethics Committees Rabin Medical Center, Tel Aviv (approval # 0778-17-RMC) and the University of Haifa (approval # 234/18). The studies were conducted in accordance with the local legislation and institutional requirements. The participants provided their written informed consent to participate in this study. Written informed consent was obtained from the individual(s) for the publication of any potentially identifiable images or data included in this article.

## Author contributions

MH: Data collection, Formal analysis, Methodology, Writing – original draft, Writing – review & editing. JC-C: Data collection, Supervision, Writing – review & editing. FA: Supervision, Writing – review & editing. KW: Data collection, Writing – review & editing.
